# Genistein is effective in inhibiting Orf virus infection *in vitro* by targeting viral RNA polymerase subunit RPO30 protein

**DOI:** 10.3389/fmicb.2024.1336490

**Published:** 2024-02-08

**Authors:** Pin Lv, Ziyu Fang, Jiyu Guan, Lijun Lv, Mengshi Xu, Xingyuan Liu, Zhuomei Li, Yungang Lan, Zi Li, Huijun Lu, Deguang Song, Wenqi He, Feng Gao, Dacheng Wang, Kui Zhao

**Affiliations:** ^1^State Key Laboratory for Diagnosis and Treatment of Severe Zoonotic Infectious Diseases, Key Laboratory for Zoonosis Research of the Ministry of Education, Institute of Zoonosis, and College of Veterinary Medicine, Jilin University, Changchun, China; ^2^College of Animal Science, Jilin University, Changchun, China

**Keywords:** Orf virus, genistein, vRNAP, drug target, viral replication

## Abstract

Orf virus (ORFV), a typical member of the genus Parapoxvirus, Poxvirus family, causes a contagious pustular dermatitis in sheep, goats, and humans. Poxviruses encode a multisubunit DNA-dependent RNA polymerase (vRNAP) that carries out viral gene expression in the host cytoplasm, which is a viral factor essential to poxvirus replication. Due to its vital role in viral life, vRNAP has emerged as one of the potential drug targets. In the present study, we investigated the antiviral effect of genistein against ORFV infection. We provided evidence that genistein exerted antiviral effect through blocking viral genome DNA transcription/replication and viral protein synthesis and reducing viral progeny, which were dosedependently decreased in genistein-treated cells. Furthermore, we identified that genistein interacted with the vRNAP RPO30 protein by CETSA, molecular modeling and Fluorescence quenching, a novel antiviral target for ORFV. By blocking vRNAP RPO30 protein using antibody against RPO30, we confirmed that the inhibitory effect exerted by genistein against ORFV infection is mediated through the interaction with RPO30. In conclusion, we demonstrate that genistein effectively inhibits ORFV transcription in host cells by targeting vRNAP RPO30, which might be a promising drug candidate against poxvirus infection.

## Introduction

Orf virus (ORFV) is a linear double-stranded DNA virus belonging to the Poxvirus family (Fleming and Mercer, 2007). It is the causative agent of Orf disease, an acute, contagious, and debilitating skin infection of sheep and goats with high morbidity, which is also called ecthyma contagiosum, contagious pustular dermatitis, or scabby mouth (Haig and Mercer, 1998). It can be transmitted to humans through direct or indirect contact with infected animals or fomites (Bala et al., 2018). Orf disease is mainly characterized by localized, proliferative, and persistent skin and oral mucosa lesions (Dal Pozzo et al., 2005). Although the lesions are normally benign, serious complications can arise from secondary infections caused by bacteria or fungi, regional lymphadenopathy, lymphangitis, erythema multiforme, bullous pemphigoid, and photoaggravated eruption. Several immune-impaired individuals develop severe progressive diseases, such as giant Orf or tumor-like lesions (Hooser et al., 1989; Smith et al., 2002; Joseph et al., 2015; Biazar et al., 2016; Lopez-Cedeno et al., 2018). Currently, antiviral treatment for ORFV infection is limited due to a lack of widely used and effective antiviral drugs (Caravaglio and Khachemoune, 2017).

Genistein (GEN) is a natural isoflavone-occurring phytoestrogen primarily found in soybeans and soybean-enriched products. Various studies have reported that genistein has a wide range of biological and pharmacological properties, including antioxidant, antiangiogenic, anti-inflammatory, anti-tumor, antibacterial, antiviral, and anthelmintic effects, and pharmacological activities on diabetes and lipid metabolism (Guo et al., 2021; Sharifi-Rad et al., 2021). Recently, genistein is recognized as potential antiviral therapeutic agent, which has been shown to be effective against several DNA and RNA viruses, including herpes simplex virus type 1, herpes B virus, cytomegalovirus, bovine herpesvirus 1, human papilloma virus, African swine fever virus, porcine reproductive and respiratory syndrome virus, human immunodeficiency virus, avian leucosis virus, and rotavirus infection (Qian et al., 2014; Sauter et al., 2014; Huang et al., 2015; Arabyan et al., 2018; LeCher et al., 2019).

In the present study, we determined that genistein was effective against ORFV infection by targeting the early stage of viral life cycles. We provided evidence that genistein exerted antiviral effect through blocking viral genome DNA transcription/replication and viral protein synthesis and reducing viral progeny. Furthermore, we identified that genistein interacted with ORFV RNA polymerase subunit RPO30 protein, a novel drug target. This may be the first report of an ORFV vRNAP RPO30 inhibitor, and genistein has great potential for developing a novel antiviral drug to treat ORFV infection.

## Materials and methods

### Cells, viruses, and drugs

Primary ovine fetal turbinate (OFTu) cells present in this study were obtained from the China Center for Type Culture Collection, Wuhan, China (CCTCC No: C2022191). Human embryo kidney 293 cells (HEK293) used in the study were purchased from CCTCC (Wuhan, China). The cell lines were maintained at 37°C in Dulbecco’s Modified Eagle Medium (DMEM) (Meilunbio, China) supplemented with 10% of fetal bovine serum (FBS) (Biolnd, Israel), 100 IU/mL penicillin, and 100 μg/mL streptomycin. The wild-type Orf virus (OV-SY17) (GenBank accession number MG712417) and the recombinant virus (termed rORFV/eGFP) expressing enhancing green fluorescent protein (eGFP) (Zhou et al., 2021) were used in the study. Genistein was purchased from Herbpurify (Chengdu, China) at 98% purity and dissolved in dimethylsulfoxide (DMSO) at a stock concentration of 20 mM.

### Gene amplication, plasmid construction, and antibody preparation

The ORFV RPO30 gene was amplified from viral genomic DNA extracted from OFTu cells infected with ORFV strain OV-SY17 and then inserted into pCMV-N-HA vector (Clontech, United States) at the EcoRI and BglII sites to generate pCMV-N-HA-RPO30 plasmid. Similarly, the ORFV RPO30 gene fragment was subcloned into the EcoRI and XhoI sites of pET32a expression vector to yield recombinant plasmid pET32a-RPO30. Site-directed mutagenesis based on the recombinant pET32a-RPO30 plasmid was performed using a Fast Mutagenesis System Kit (Transgen Biotech, China) to obtain two mutants (L107A and R75A). The primers used for plasmid construction are presented in Table 1. The pCMV-N-HA-RPO35 recombinant plasmid was constructed in our laboratory. Anti-RPO30 polyclonal antibody used in the study was prepared in our laboratory.

**Table 1:** Sequences of the oligonucleotide primers used in the study.

Primers	Sequence (5′-3′)
RRO30-HA-Fw	CCATGGAGGCCCGAATTCGGATGGACGAAGACCGGCTGCGCGACC
RPO30-HA-Rv	CGCGGTACCTCGAGAGATCTTTAGGATTTTGGCTTGCCTTTCTTC
RPO30-pET32a-Fw	CTGATATCGGATCCGAATTCATGGACGAAGACCGGCTGCGCGACC
RPO30-pET32a-Rv	TGGTGGTGGTGGTGCTCGAGGGATTTTGGCTTGCCTTTCTTCTCG
RPO30-L107A-Fw	GCGCTGCGGTACCTGGCCTTCGCGGTC
RPO30-L107A-Rv	GCCAGGTACCGCAGCGCGTCGTGCTCC
RPO30-R075A-Fw	CTCGTACAAGAACAAGGCCAGCCTGGAG
RPO30-R075A-Rv	GCCTTGTTCTTGTACGAGAGCCGGTTC
GADPH-Fw	TTATGACCACTGTCCACGCC
GADPH-Rv	TCAGATCCACAACGGACACG
ORFV011(B2L)-Fw	CAATGATCTGCGGCCAGTAC
ORFV011(B2L)-Rv	AACTTCCACCTCAACCACTCC
ORFV042-Fw	TCCCGATCGTGTGCTCAAC
ORFV042-Rv	CACCGTCCGCAAAACAACC
ORFV059-Fw	AGGTATGCCAGGATGAAGATG
ORFV059-Rv	AAGCCCGAGATGGTAAAGC
ORFV069-Fw	GGCGAGCTCATGTTCCTCTT
ORFV069-Rv	TAGTCCTCCAGCGAGACGAT
ORFV070-Fw	ATGCGCGAGTACCTCTACAAG
ORFV070-Rv	AGGCAGTACTTGGACTCGTTG
ORFV072-Fw	CCTGTGCTCGTACTTGGTC
ORFV072-Rv	CTTGGAGAGCACGGACTTGT

### Cytotoxicity assay

OFTu cells were, respectively, treated with medium containing different concentrations of genistein (0–600 μM) for 72 h. Cell viability was measured using Cell Counting Kit-8 assay (Meilunbio, China), according to the manufacturer’s instructions. The 50% cytotoxic concentration (CC_50_) was calculated using GraphPad Prism 8 software (GraphPad, Inc., United States).

### *In vitro* antiviral activity assay

The antiviral effects of genistein with different concentrations (3.12, 6.25, 12.5, 25, 50, and 100 μM) against ORFV infection were determined using confluent cultures of OFTu cells. For antiviral effect assay, OFTu cells grown in 24-well cell culture plate (2 × 10^5^ cell/well) were incubated with a mixture of 10^5.3^ TCID_50_/well virus. After incubation at 37°C for 1 h, the virus inoculum was removed and replaced by maintenance medium containing various concentrations of genistein. The cell monolayer was incubated at 37°C for another 72 h and then collected. Virus titer was determined by the 50% tissue culture infectious dose (TCID_50_) assay.

### Time-of-addition

The mode of genistein during the viral life cycle was tested using the time-of-addition assay. In brief, the genistein was added to the cell cultures at 1 h prior infection (pre-treatment), 0 h (simultaneous-treatment), and 1 h post infection (post-treatment). Infected and untreated cells were served as controls. Virus titer was determined by TCID_50_ assay at 72 hpi.

### Virucidal assay

The virus suspension containing 10^5.3^ TCID_50_/well virus was incubated with varying concentrations of genistein (0 μM, 50 μM, and 100 μM) (v/v) for 1 h at 37°C prior to the infection. Then, the mixture was used to infect the confluent monolayer OFTu cells. After incubation at 37°C for 1 h, the inocula were removed; cells were washed three times with PBS and replaced by DMEM medium containing 2% FBS. Virus was titrated by TCID_50_ assay at 72 hpi.

### Attachment and penetration assays

For the attachment assay, the monolayer OFTu cells grown in 24-well cell culture plate (2 × 10^5^ cell/well) were co-incubated with genistein and ORFV (10^6.3^ TCID_50_) at 4°C for 1 h. The unbound virus was removed; cells were thoroughly washed with cold PBS and replaced by DMEM medium containing 2% FBS. For the penetration assay, the monolayer OFTu cells were incubated with ORFV (10^6.3^ TCID_50_) at 4°C for 1 h to allow virus adsorption. The unbound virus was removed, and cells were incubated with genistein at 37°C for 1 h. At the end of the incubation, the cells were washed with PBS and then cultured for 72 h in the maintenance medium. Virus titer was determined by TCID_50_ assay.

### Indirect immunofluorescence assay

OFTu cells grown in 24-well cell culture plate (2 × 10^5^ cell/well) were infected with the rORFV/eGFP (10^5.3^ TCID_50_). After an adsorption period of 1 h, the virus inoculum was removed and replaced by DMEM maintenance medium containing 100 μM genistein. After 48 h of infection, the cell samples were collected and washed three times with PBS. Subsequently, Hoechst 33342 (Thermo scientific, United States) at the concentration of 1 μg/mL was added and incubated for nuclear fluorescence staining at 37°C for 5 min. Finally, the cells were washed and observed under a fluorescence microscope.

### Quantification PCR assay

The viral DNA copies in OV-SY17-infected OFTu cells mock-treated or treated with 100 μM of genistein for 24 h were determined by qPCR using the LineGene 9,600 Series (Bioer Technology, China). Amplification was performed in 10 μL reaction volumes: 0.5 μL of 10 μM of each primer (Table 1), 5 μL of 2 × SYBR Green qPCR Master Mix (Bimake, United States), and 0.5 μL of template DNA and nuclease-free sterile water to a final volume of 10 μL. The thermal cycling conditions were an initial denaturation step of 95°C for 2 min, followed by 40 cycles of 95°C for 15 s and 60°C for 60 s. All reactions were performed in triplicate. A melt curve analysis was enabled at the end of amplification. The DNA copies were quantified and calculated based on the standard curves of pET-32a-B2L plasmid (Wang et al., 2022).

Furthermore, the transcriptional analysis of ORFV early, middle, and late genes in OV-SY17-infected OFTu cells mock-treated or treated with 100 μM of genistein for 24 h was performed using qRT-PCR. In brief, the total RNA was extracted from infected cells at different time points of infection using RNAiso Plus (Takara, China). First-strand cDNA was synthesized from 3 μg of total RNA, using M-MLV (Takara, China). The real-time PCR reaction system contained 0.5 μL of template cDNA, 0.5 μL of 10 μM of each primer (Table 1), 5 μL of 2 × SYBR Green qPCR Master Mix (Bimake, United States), and nuclease-free sterile water to a final volume of 10 μL. The thermal cycling conditions were an initial denaturation step of 95°C for 2 min, followed by 40 cycles of 95°C for 15 s and 60°C for 60 s. qRT-PCR analysis was performed using the LineGene 9,600 Series (Bioer Technology, China), and the mRNA levels of viral genes and the reference gene GADPH were quantified using the 2-△△Ct method. Data were normalized against GAPDH.

### Western blotting

OFTu cells mock-infected or infected with OV-SY17 (MOI = 1) and treated with different concentrations of genistein (0 μM, 10 μM, 50 μM, and 100 μM) were collected, washed twice with PBS, and then lysed on ice for 30 min using RIPA lysis buffer (Beyotime, China) containing 1% protease inhibitors. Protein samples were subjected to quantification by a BCA assay (Beyotime, China) prior to Western blot analysis. The equal amounts of protein samples were resolved by SDS-PAGE on 10% gels and then transferred to a PVDF membrane (Millpore, United States). The membranes were blocked in 5% non-fat milk and incubated overnight at 4°C with primary antibodies, such as ORFV059 polyclonal antibody (prepared in our laboratory) or β-actin monoclonal antibody (Proteintech, China). Subsequently, the membranes were incubated with appropriate secondary antibodies. Protein bands were visualized using ECL Western blotting detection reagents. The results were normalized to those for β-actin.

### Cellular thermal shift assay

The cellular thermal shift assay (CETSA), as described previously (Guo et al., 2020), was employed to evaluate the binding of genistein and viral RPO30 protein in living cells. In brief, HEK293 cells were, respectively, transfected with pCMV-N-HA-ORFV RPO30 or pCMV-N-HA-ORFV RPO35 plasmid using LipoFiter 3.0 (Hanbio, China) for 40 h and exposed to 100 μM genistein or DMSO at 37°C with 5% CO_2_ for 2 h. Subsequently, the cells were washed with PBS to remove excess drugs, harvested, and then lysed on ice for 30 min in RIPA lysis buffer. Protein concentrations were determined by the BCA protein quantification assay (Beyotime, China). Protein solutions were adjusted to 1 mg/mL, and 30 μL aliquots of supernatants were added to a Thermal Cycler (Bio-Rad, USA), heated at different temperatures for 5 min, and then chilled at room temperature for 3 min. After centrifugation at 13,000×*g* for 30 min at 4°C, supernatants were transferred to new tubes, separated by SDS-PAGE on 10% gels, transferred to PVDF membrane, and incubated with primary antibodies HA tag rabbit polyclonal antibody (Proteintech, China) or GAPDH/β-actin mouse monoclonal antibody (Proteintech, China) and appropriate secondary antibodies.

### Localized surface plasmon resonance

The interaction between genistein and ORFV RPO30 protein was determined using an OpenSPR localized surface plasmon resonance (LSPR) instrument (Nicoya, Canada) at 25°C, as described previously (Yi et al., 2018; Du et al., 2021). In brief, viral RPO30 proteins were captured on a COOH chip using a standard amine coupling system. The affinity between different concentrations of genistein and ORFV RPO30 protein was measured using HEPES (pH 7.4) buffer with 1% DMSO as running buffer at a flow rate of 20 μL/min for an association phase of 240 s, followed by 360 s of dissociation. The kinetic parameters of the binding reaction were calculated and visualized using TraceDrawer software (Yang et al., 2011).

### Molecular modeling of ORFV RPO30 protein–genistein interactions

To date, no crystal structure of ORFV RPO30 protein is available, and the atomic structure of the viral RPO30 protein based on the poxvirus transcription initiation complexes and DNA-directed RNA polymerase 30 kDa polypeptide (Protein Data Bank ID:7aoh.1. K; resolution, 2.70 Å) was built on the Swiss model website for molecular modeling. The structure of genistein was determined using the PubChem database (CCDC Number: 5280961). A molecular docking study was conducted to investigate the binding mode and amino acid interactions between genistein and ORFV RPO30 protein using AutoDock Vina 1.1.2 (Trott and Olson, 2010). The default parameters were used unless otherwise indicated. After the docking simulation, the best-docked pose of the ORFV RPO30-genistein complexes was used to perform a 40 ns molecular dynamics (MD) simulation using The Amber 14 and AmberTools 15 programs (Salomon-Ferrer et al., 2013).

### Fluorescence quenching assay

The binding constant (KA) between genistein with ORFV RPO30 protein and its mutants was measured using a fluorescence quenching method as described previously (Bandyopadhyay et al., 2002; Bodenreider et al., 2009). In brief, the purified ORFV RPO30 protein was diluted to 0.8 mg/mL. Then, 2 μL of diluted protein solution was, respectively, mixed with different concentrations of genistein (0–50 μM). For the fluorescence measurement, a 280-nm excitation wavelength with a 5-nm bandpass was used. The fluorescence emission spectrum was scanned and recorded at 290 nm and 400 nm, respectively. All measurements were carried out thrice, and the average value of the fluorescence intensity changes was used in the calculations. The binding curves and KA values were analyzed according to the descriptions previously reported (Kim et al., 2006).

### Statistical analysis

The data of each group in the individual experiments were expressed as the mean ± SD and analyzed by GraphPad Prism 8.0. Statistical significance was accepted as *p*-values<0.05.

## Results

### Genistein’s inhibitory effect on Orf virus replication

**Figure 1:**
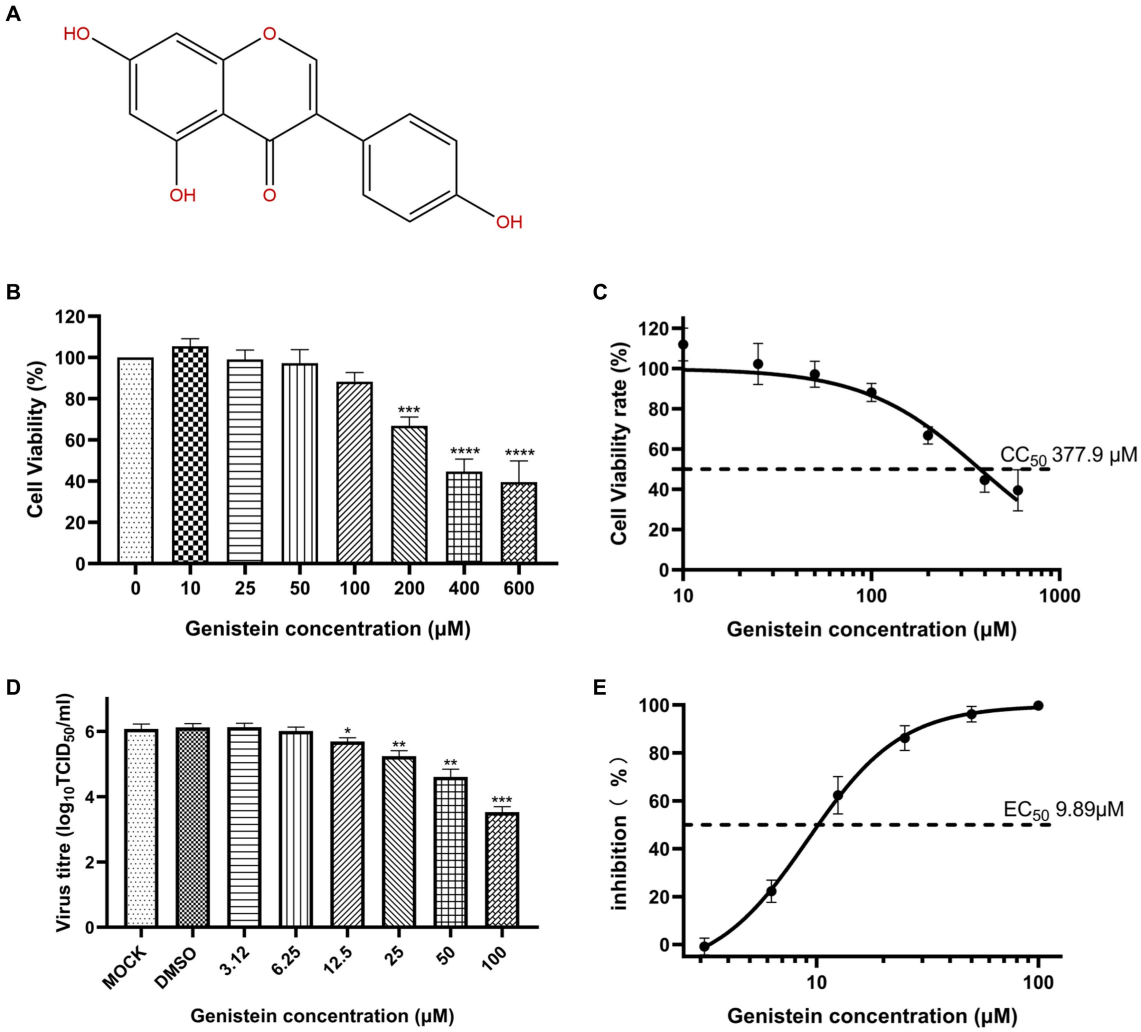
The antiviral activity of genistein against ORFV in OFTu cells. **(A)** Chemical structure of genistein. **(B)** The cytotoxicity of genistein in OFTu cells was measured by standard CCK8 assay. The cell viability rate was calculated using the following formula: cell viability (%) = (Optical Density (OD) value of drug-treated group-OD value of blank)/(OD value of control group-OD value of blank) × 100%. Each value represents the average of three independent experiments. **(C)** The 50% cytotoxic concentration (CC_50_) was calculated using a non-linear regression analysis. **(D)** *In vitro* antiviral activity of genistein. The antiviral effects of genistein with different concentrations (3.12, 6.25, 12.5, 25, 50, and 100 μM) on ORFV replication in OFTu cells were investigated, and the virus titers were determined by TCID_50_ assays. Virus titers were markedly reduced in genistein-treated OFTu cells with increasing drug concentrations ranging from 12.5 μM to 100 μM in a dose-dependent manner. **(E)** The concentration for 50% of maximal effect (EC_50_) value of genistein was calculated by virus yield in different concentrations of genistein. Statistical significance is denoted by * *p* < 0.05, **p < 0.01, ***p < 0.001, and ****p < 0.0001.

The chemical structure of genistein is shown in Figure 1A. The cytotoxicity of genistein was measured using CCK8 assay after OFTu cells incubated with genistein for 72 h. As shown in Figures 1B,C, OFTu cell proliferation was significantly inhibited by genistein at concentrations above 200 μM with the CC_50_ value of 377.9 μM. To determine the antiviral activity of genistein against ORFV, OFTu cells infected with OV-SY17 were incubated with genistein at different concentrations ranging from 3.12 μM to 100 μM. As shown in Figures 1D,E, virus titers were markedly reduced in genistein-treated OFTu cells with increasing drug concentrations ranging from 12.5 μM to 100 μM in a dose-dependent manner, indicating that genistein has antiviral activity against ORFV, with the concentration for 50% of maximal effect (EC_50_) value of 9.89 μM. At 100 μM concentration, the viral titer in untreated control (6.12 ± 0.12 log TCID_50_/mL) reduced to 3.5 ± 0.17 TCID_50_/mL (*** *p* < 0.001). No significant antiviral effect was detected in ORFV-infected cells treated with genistein at concentrations of less than 6.25 μM.

### Inhibitory effect of genistein on viral DNA, RNA, and protein synthesis

**Figure 2:**
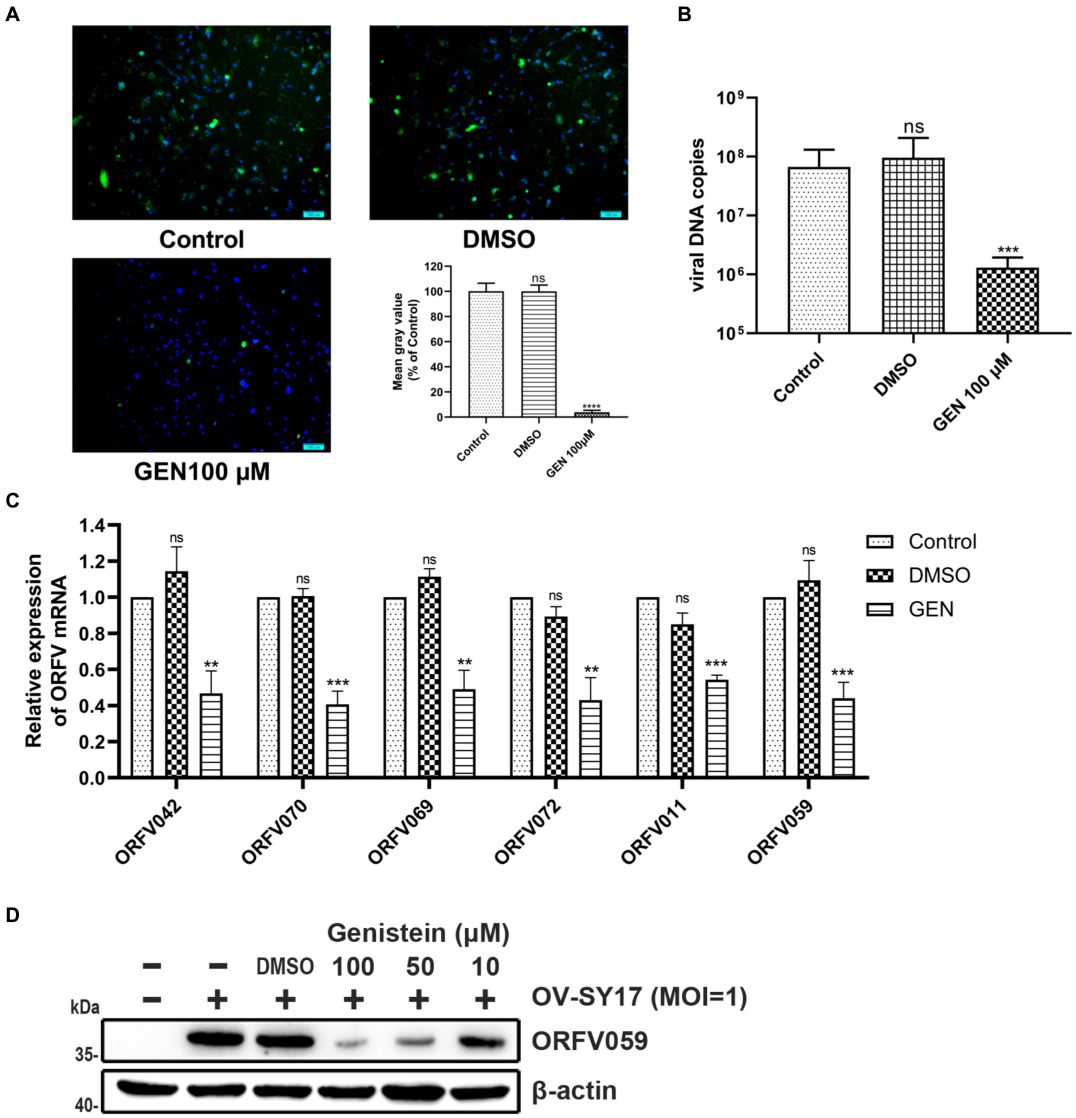
Inhibitory effect of genistein on viral yield, viral DNA replication, and viral protein synthesis. **(A)** The fluorescence intensity of eGFP in OFTu cells infected with rORFV/eGFP (MOI = 1) treated or untreated with genistein was observed under fluorescent microscopy. The y-axis represents the intensity of the signal percentage normalized against the control. EGFP expression (green) for the monitoring of viral infection and Hoechst 33342 (blue) for nuclear staining. Scale bar, 100 μm. **(B)** At 24 h post infection, the viral DNA copies in OV-SY17-infected OFTu cells treated or untreated with genistein were determined by qPCR. When OV-SY17-infected cells exposed to 100 μM genistein, it provoked a significant reduction in the amounts of viral DNA (****p < 0.0001). **(C)** Transcriptional analysis of ORFV early genes ORFV042/ORFV070, middle genes ORFV069/ORFV072, and late genes ORFV011/ORFV059 in OV-SY17-infected OFTu cells treated or untreated with genistein was performed by qRT-PCR. Data are normalized against GAPDH and are presented relative to mock-treated cells. **(D)** Viral protein synthesis was detected by Western blotting using anti-ORFV059 polyclonal antibody. β-actin was used as a loading control. Molecular weights (kDa) of evaluated proteins are indicated on the left of immunoblot image. For Western blotting, genistein was, respectively, used at 10, 50, and 100 μM concentrations in OFTu cells infected with OV-SY17 (MOI = 1). Values represent mean and standard deviation results from three independent experiments. Statistical significance is denoted by *p < 0.05, **p < 0.01, ***p < 0.001, and ****p < 0.0001.

To further investigate the antiviral effect of genistein against ORFV *in vitro*, OFTu cells infected with rORFV/eGFP (Zhou et al., 2021) were treated with or without genistein, and then, the fluorescent intensity of eGFP in infected cells was observed at 48 hpi. Compared with the untreated group, the GFP fluorescence signal showed significant attenuation in rORFV/eGFP-infected cells treated with genistein. The infection rate of rORFV/eGFP was significantly reduced by up to 96.14% (****p < 0.0001) (Figure 2A) in the genistein-treated group, indicating that genistein could inhibit rORFV/eGFP yield in OFTu cells. At 24 h post infection, the viral DNA copies in OV-SY17-infected OFTu cells treated or untreated with genistein were determined by the fluorescent quantitative PCR. As shown in Figure 2B, when OV-SY17-infected cells exposed to 100 μM genistein, it provoked a significant reduction in the amount of viral DNA (****p < 0.0001). The qPCR results at 24 h post-infection indicated that genistein could inhibit viral production rather than the DNA replication cycle, which could serve as a confirmation for TCID_50_ titration. Furthermore, the transcript levels of the early viral genes ORFV 042/ORFV070, the middle viral genes ORFV069/ORFV072, and the late viral genes ORFV011/ ORFV 059 were detected by qRT-PCR. The transcript levels of these viral genes in OV-SY17-infected cells treated with genistein showed significant reduction from 24 hpi onward (Figure 2C). In addition, viral protein synthesis was detected by Western blotting using self-prepared anti-ORFV059 polyclonal antibody. As shown in Figure 2D, the expression of ORFV059 protein showed obvious decrease in OV-SY17-infected cells treated with genistein with increasing drug concentrations in a dose-dependent manner.

### Genistein targets the viral transcription stage

**Figure 3:**
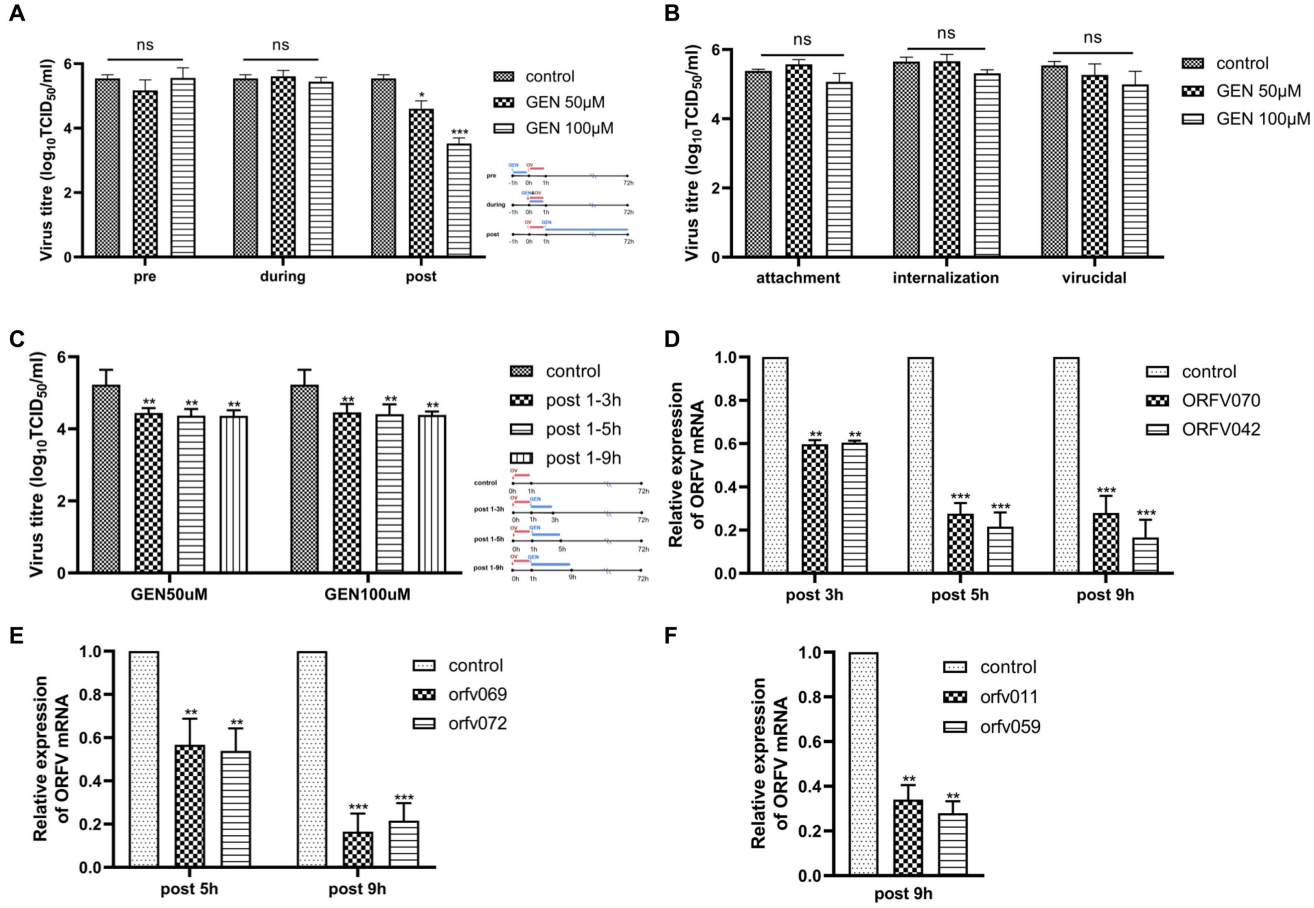
Evaluation of the antiviral activity of genistein on specific step of the ORFV life cycle. **(A)** A time-of-addition assay was performed to determine the key step of ORFV infection targeted by genistein. In brief, OFTu cells were treated with genistein for 1 h prior to infection (pre-treatment), 0 h (simultaneous treatment), or 1 h post infection (post-treatment). Virus titers were determined by TCID_50_ assay. **(B)** Effect of genistein on virion attachment, internalization during ORFV entry, and extracellular viral particles. **(C)** Antiviral effect of genistein on the duration of ORFV transcription including the early, middle, and late stages. The concentrations of genistein used in **(A–C)** were 50 and 100 μM, respectively. Infected cells were treated with DMSO-containing medium as a control. **(D–F)** The transcriptional analysis of ORFV early genes (ORFV 042 and ORFV 070) **(D)**, middle genes (ORFV 069 and ORFV072) **(E)**, and late genes (ORFV 011 and ORFV059) **(F)** in OV-SY17-infected OFTu cells mock-treated or treated with of 100 μM genistein detected through qRT-PCR. Relative transcription levels of the indicated genes were normalized against GAPDH and were presented relative to mock-treated cells. Values represent mean and standard deviation results from three independent experiments. Statistical significance is denoted by *p < 0.05, **p < 0.01, ***p < 0.001, ****p < 0.0001.

To understand the underlying antiviral mechanism of genistein, a time-of-addition assay was performed to determine the step of ORFV infection targeted by genistein. OFTu cells were treated with genistein at 1 h prior to infection (pre-treatment), 0 h (simultaneous treatment), or 1 h post infection (post-treatment). Virus titers were determined by TCID_50_ assay. As shown in Figure 3A, genistein showed the strong anti-ORFV activity in post-treatment with genistein. The viral titer was significantly reduced by 2.01 log (> 95%, *** *p* < 0.001). To further determine which stages of the ORFV life cycle are inhibited by genistein upon ORFV infection, we examined the effect of genistein on virion attachment, internalization during ORFV entry, and extracellular viral particles. As shown in Figure 3B, genistein at the concentrations of 50 μM and 100 μM did not interfere with viral entry, and no virucidal effect against ORFV by genistein was observed. In post-treatment assay, OFTu cells were infected with ORFV, and then, the virus inoculum was removed at 1 h post infection. To further determine whether genistein was involved in regulating the early, middle, and late stages of viral transcription, genistein was added to infected cells at the concentrations of 50 μM and 100 μM, respectively. At 3 h, 5 h, and 9 h post infection, genistein was removed from culture medium. Subsequently, the treated cells were rested for 3 h and then harvested for TCID_50_ assay. The data, as shown in Figure 3C, suggested that the viral titers were markedly reduced in all treatment groups (** *p* < 0.01), compared with those in the virus control, by genistein at a concentration of 100 μM. Transcriptional analysis of ORFV early genes ORFV042/ORFV070, middle genes ORFV069/ORFV072, and late genes ORFV011/ORFV059 showed that the viral transcription activity was significantly reduced in OV-SY17-infected OFTu cells treated with genistein at the concentrations of 100 μM (** *p* < 0.01, *** *p* < 0.001) (Figures 3D–F). Based on the above results, genistein might exert its antiviral effects by inhibiting the transcription stage of ORFV life cycle.

### Genistein interacts with ORFV RNA polymerase RPO30 protein

**Figure 4:**
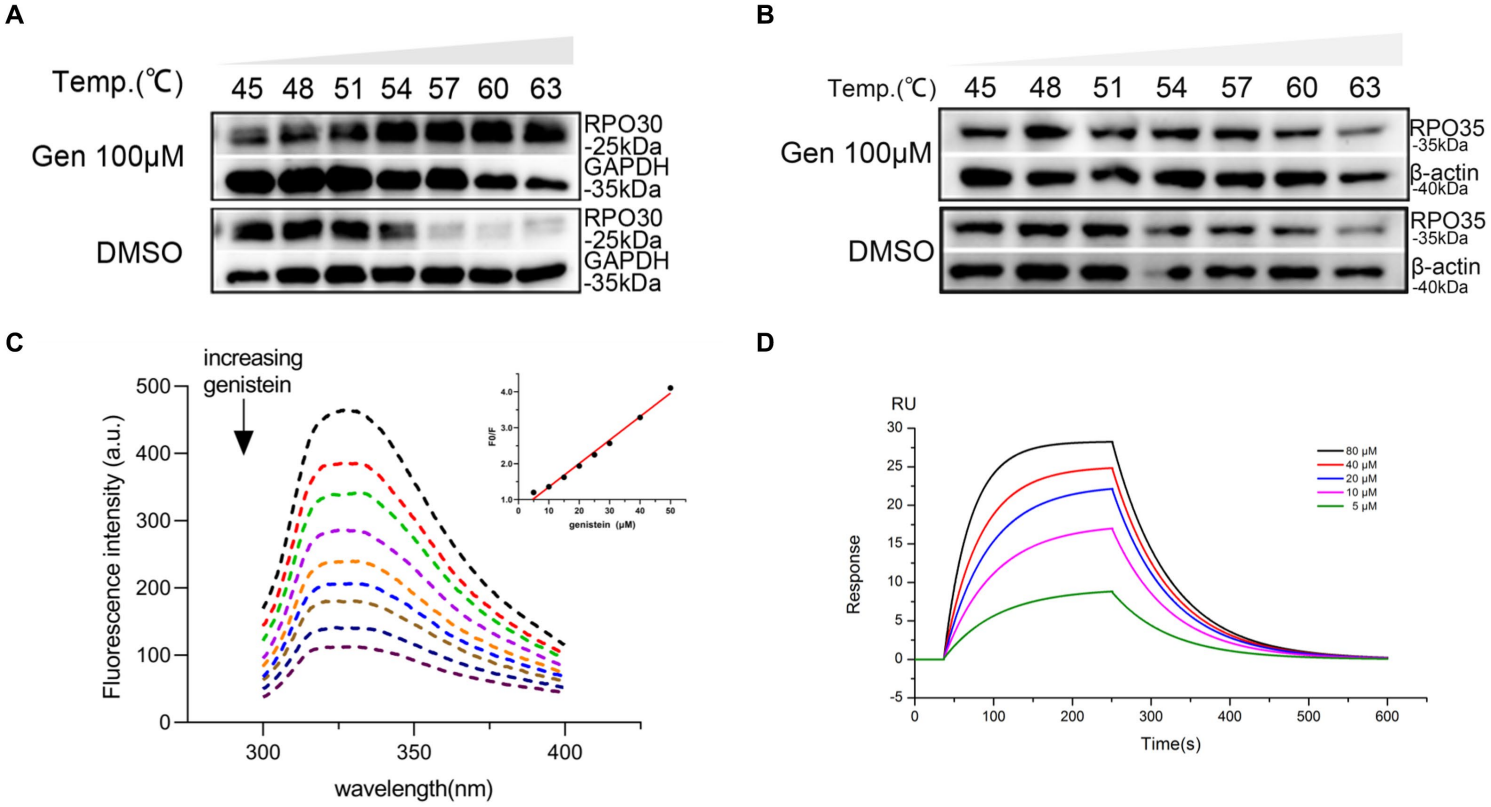
Genistein interacts with ORFV RNA polymerase RPO30 protein. **(A)** A cellular thermal shift assay (CETSA) was performed to determine the binding of genistein to the subunits of ORFV RNA polymerase involved in viral transcription. The results of immunoblotting assays showed the thermostability of the RPO30 subunit of ORFV RNA polymerase following heating in the presence (+) or absence (−) of genistein at the concentration of 100 μM. **(B)** The binding of genistein to the RPO35 subunit was determined using CETSA assay. The results of immunoblotting assays showed the thermostability of the RPO35 subunit of ORFV RNA polymerase following heating in the presence (+) or absence (−) of genistein at the concentration of 100 μM. **(C)** The binding affinity between genistein and the RPO30 subunit of ORFV RNA polymerase was measured by a fluorescence quenching analysis. The fluorescence emission spectra of the RPO30 subunit of ORFV RNA polymerase in the presence of various concentrations of genistein at λex = 280 nm. Inset: Stern–Volmer plot described the RPO30 subunit of ORFV RNA polymerase quenching caused by interacting with genistein. **(D)** LSPR experiment was performed to further verify the binding affinity of genistein with the RPO30 subunit of ORFV RNA polymerase.

To evaluate the binding potency of genistein to ORFV RNA transcription-related proteins in living cells, cellular thermal shift assay (CETSA) was performed to determine the binding of genistein to the subunits of ORFV RNA polymerase involved in viral transcription. The effect of genistein on the thermostability of the RPO30 subunit of ORFV RNA polymerase is presented in Figure 4A. The results indicated that the presence of genistein at the concentration of 100 μM in HEK293 cells significantly increased the stability of the RPO30 subunit of ORFV RNA polymerase at 57, 60, and 63°C. Aside from RPO30, we also investigated the effect of genistein on the thermostability of the RPO35 subunit. We found that genistein treatment could not prevent the degradation of RPO35 subunit following heating, which indicated that there was no interaction between genistein and RPO35 subunit (Figure 4B). To further determine the binding affinity between genistein and the RPO30 subunit of ORFV RNA polymerase, a fluorescence quenching analysis and LSPR experiment were performed. As shown in Figure 4B, with the increase in genistein concentration, the fluorescence of the RPO30 subunit of ORFV RNA polymerase was gradually quenched, and the fluorescence intensity was linear with the concentration of genistein (Figure 4C, inset). The binding constant (KA) value of the purified RPO30 to genistein was 3.13×10^4^ L/mol, suggesting that genistein interacted with the RPO30 subunit of ORFV RNA polymerase. Additionally, the LSPR experiment results further revealed that genistein interacted directly with the RPO30 subunit in a dose-dependent manner, with a KD of 6.68 × 10^−5^ mol/L (Figure 4D).

### Determination of the molecular mechanism of the interaction between genistein and RPO30

**Figure 5:**
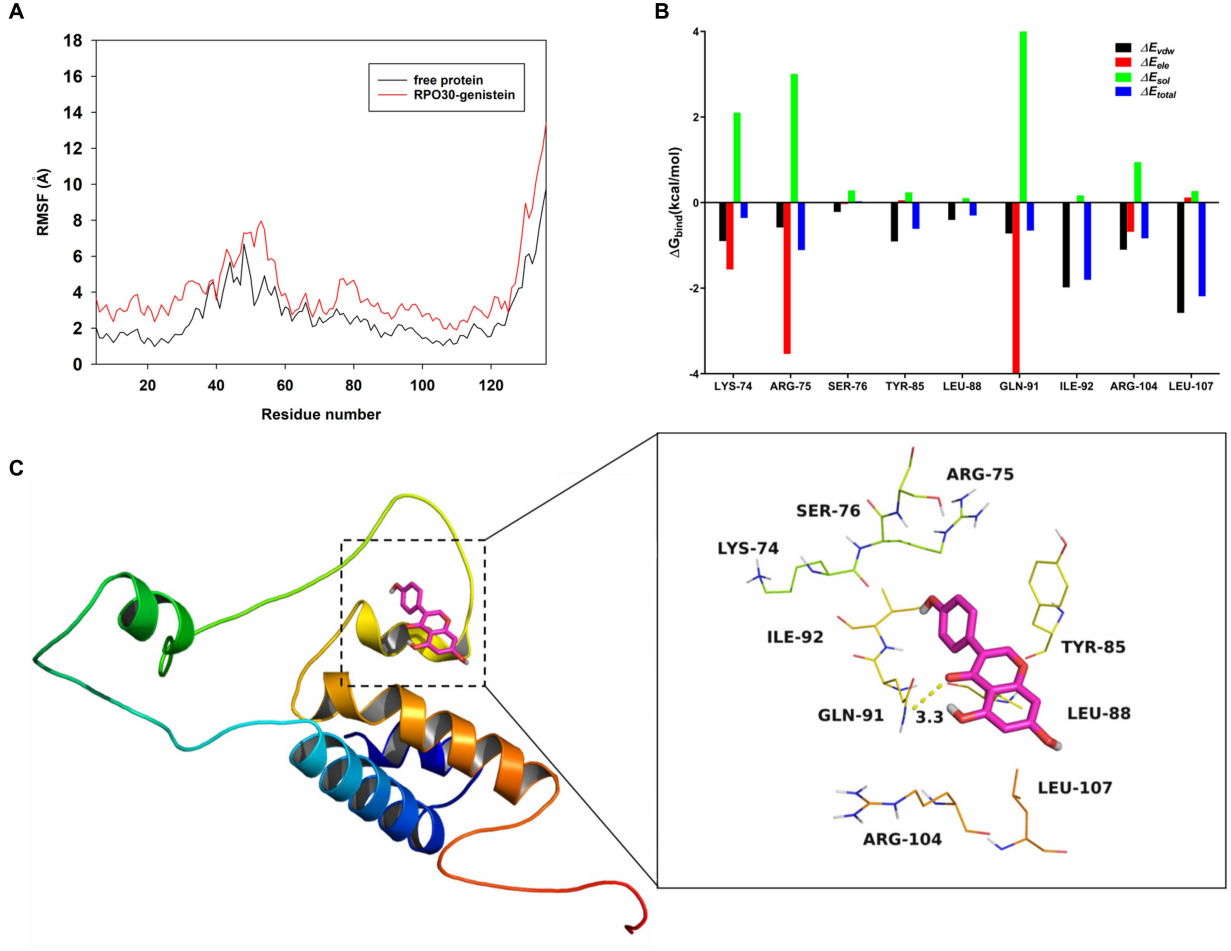
Molecular modeling for the interaction between genistein and ORFV RPO30 subunit. **(A)** RMSF (Å) graph of free-ORFV RPO30 (black), and the ORFV RPO30-genistein complex (red) during 40-ns MD. **(B)** Docking model of genistein with the RPO30 subunit of ORFV RNA polymerase during a molecular dynamics simulation. **(C)** Binding free energy decomposition in each residue between genistein and the modeled ORFV RPO30 subunit.

Through the root mean square fluctuations (RMSF), we identified that RPO30 exhibited different flexibility in binding sites with or without genistein (Figure 5A), implying that the flexibility residues might play a key role in the recognition of potential binding sites during RPO30-genistein interactions. Furthermore, the molecular modeling studies were conducted to explore the binding domain of the RPO30-genistein complex using an *in silico* model, which was found that the Arg-75 residue provided a strong electrostatic (∆*E_ele_*) contribution, with ∆*E_ele_* < −3.5 kcal/mol (Figure 5B). Detailed analysis showed that the residue Arg-75 was close to the phenyl group of the genistein, forming the cation-π interaction (Figure 5C). Additionally, the residue Leu-107 with the ∆*E_vdw_* of < −2.5 kcal/mol (Figure 5B) indicated an appreciable *Van der Waals* interaction with genistein because of the close proximity between the residue and genistein. Except for the residue Leu-107, the majority of the decomposed energy interaction originated from *Van der Waals* interactions, apparently through hydrophobic interactions (i.e., Leu-88 and Ile-92). Furthermore, the total binding free energy for the RPO30-genistein complex calculated according to the MMGBSA approach, and an estimated ∆G*_bind_* of −10.2 kcal/mol was found for genistein, suggesting that genistein can strongly bind to and interact with the binding site of RPO30.

In light of the predictions by molecular modeling, two RPO30 subunits of ORFV RNA polymerase mutants, L107A-RPO30 and R75A-RPO30, were generated via site-directed mutagenesis. Then, the binding affinity between genistein and the purified RPO30 subunit mutants was investigated using a fluorescence quenching assay. As shown in Table 2, the KA value of the purified RPO30 to genistein was 3.13×10^4^ L/mol, whereas a reduction in KA value was observed in RPO30 subunit mutants, L107A-RPO30 and R75A-RPO30, to genistein. These results suggested that the amino acid residues, Leu107 and Arg75, were the main residues involved in the binding of genistein to the RPO30 subunit of ORFV RNA polymerase.

**Table 2:** The values of the binding constants (KA) based on the fluorescence quenching assay.

Proteins	WT-ORFV RPO30	L107A	R75A
KA (1 × 10^4^) L/mol	3.13	1.56	2.41
*n*	1.078	0.8571	0.9693

### The involvement of genistein-RPO30 interaction in the inhibition mediated by genistein

**Figure 6:**
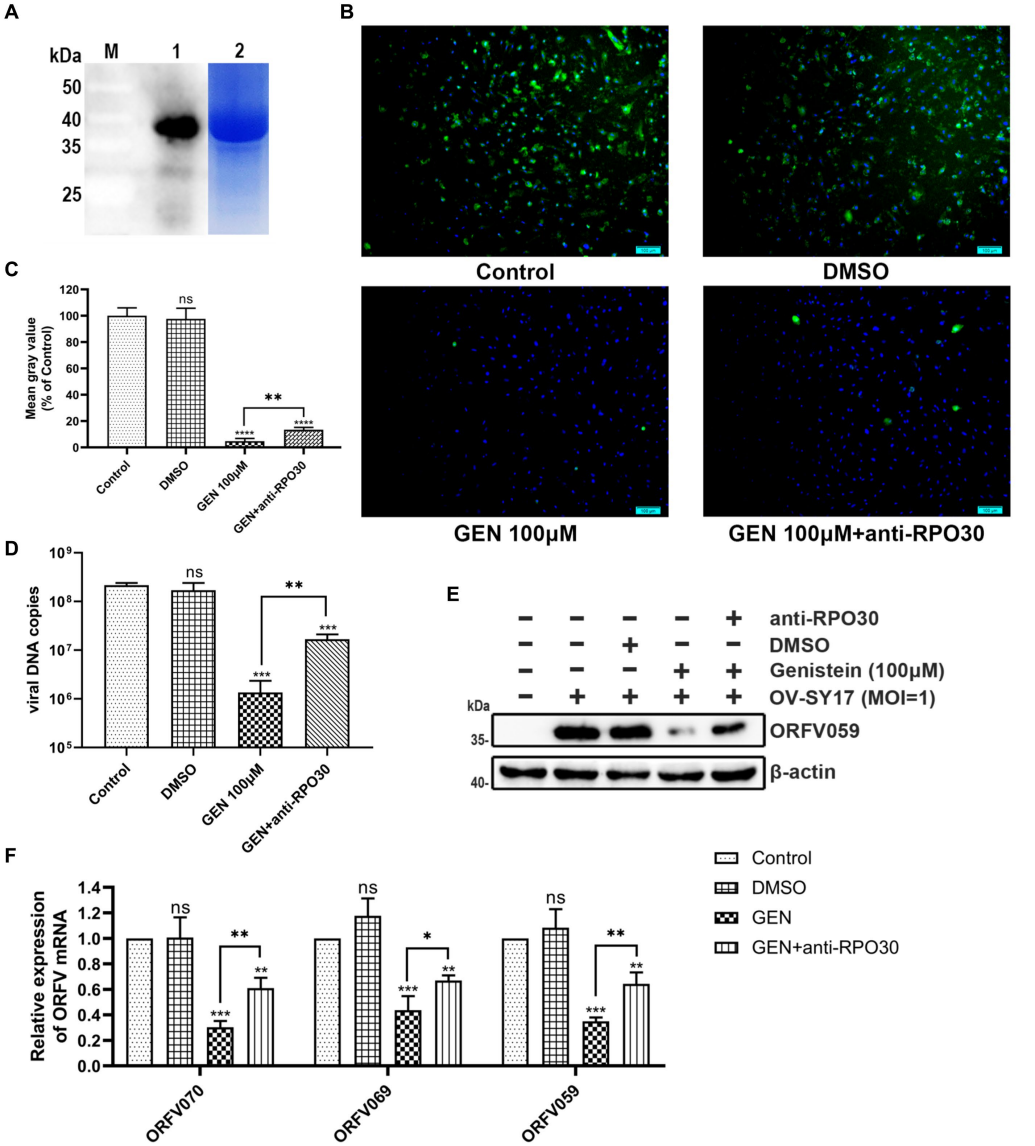
The inhibitory effect against ORFV infection exerted by genistein is mediated through the interaction with ORFV RPO30. **(A)** M: Protein Marker; Lane 1: Anti-ORFV059 polyclonal antibody was detected using the purified RPO30 protein; Lane 2: The purified RPO30 protein. **(B)** The fluorescence intensity of eGFP in OFTu cells infected with rORFV/eGFP (MOI = 1) treated with genistein after culturing in the presence or absence of antibody against RPO30 was observed under fluorescent microscopy. EGFP expression (green) for the monitoring of viral infection, and Hoechst 33342 (blue) for nuclear staining. Scale bar, 100 μm. **(C)** The rORFV/eGFP yield in the RPO30 blocking or unblocking group by fluorescence density analysis assay. **(D)** The viral DNA copies in OV-SY17-infected OFTu cells treated with genistein after culturing in the presence or absence of antibody against RPO30 were determined by the fluorescent quantitative PCR. When blocking RPO30 in OV-SY17-infected cells exposed to genistein, it provoked a significant increase in the amounts of viral DNA (**p < 0.01). **(E)** Viral protein synthesis was detected by Western blotting using anti-ORFV059 polyclonal antibody. β-actin was used as a loading control. Molecular weights (kDa) of evaluated proteins are indicated on the left of immunoblot image. For Western blotting, genistein was used at 100 μM concentration in OFTu cells infected with OV-SY17 (MOI = 1) culturing in the presence or absence of antibody against RPO30. **(F)** Transcriptional analysis of ORFV early, middle, and late genes in the RPO30 blocking or unblocking group were performed by qRT-PCR. Data are normalized against GAPDH and are presented relative to mock-treated cells. Values represent mean and standard deviation results from three independent experiments. Statistical significance is denoted by *p < 0.05, **p < 0.01, ***p < 0.001, and ****p < 0.0001.

To further determine whether the inhibitory effect exerted by genistein against ORFV infection is mediated through the interaction with RPO30, OFTu cells were infected with rORFV/eGFP treated with genistein after culturing in the presence or absence of antibody against RPO30. When blocking RPO30 by using RPO30 antibody (Figure 6A) in rORFV/eGFP treated with genistein, the fluorescence intensity showed significant increase in rORFV/eGFP-infected cells treated with genistein (Figure 6B). The rORFV/eGFP yield was significantly increased in the RPO30 blocking group (Figure 6C), indicating that the inhibition exerted by genistein was reduced. Subsequently, the viral DNA copies in OV-SY17-infected OFTu cells treated with genistein after culturing in the presence or absence of antibody against RPO30 were determined by the fluorescent quantitative PCR. As shown in Figure 6D, when blocking RPO30 in OV-SY17-infected cells exposed to genistein, it provoked a significant increase in the amounts of viral DNA (**p < 0.01). The results suggested that the viral DNA synthesis was increased in the presence of antibody against RPO30. Furthermore, the transcript levels of the early viral genes ORFV070, the middle viral genes ORFV069, and the late viral genes ORFV 059 were detected by qRT-PCR. The transcript levels of these viral genes in the RPO30 blocking group showed significant increase from 24 hpi onward (Figure 6F). In addition, viral protein synthesis was detected by Western blotting using self-prepared anti-ORFV059 polyclonal antibody. As shown in Figure 6E, the expression of ORFV059 protein showed obvious increase in the RPO30 blocking group. The results indicated that RPO30 is involved in the inhibitory effect against ORFV infection by genistein.

## Discussion

Orf is a globally neglected zoonotic disease, so its incidence is greatly underestimated (Bohelay and Duong, 2019; Kassa, 2021; Gaspari et al., 2022). The use of conventional vaccines to prevent ORFV infections is debatable due to its short-term protection; however, there is no promising vaccine candidate available to prevent ORFV infections (Ganter, 2015). Thus, the development of effective drugs remains of paramount importance to control Orf. Currently, cidofovir [(S)-1-(3-hydroxy-2-phosphonylmethoxypropyl)cytosine [HPMPC], a nucleoside analog, has been demonstrated to have inhibitory effects on the replication of multiple orthopoxviruses and parapoxviruses *in vitro*. It can be administered intravenously but not orally bioavailable (Nettleton et al., 2000). Additionally, nephrotoxicity is a major side effect and a dose-limiting factor for cidofovir (Ortiz et al., 2005), which further limited its utility as an effective antipoxvirus therapeutic agent. Tecovirimat (ST-246) is an orally bioavailable antipoxvirus compound, which exhibits selective activity against multiple orthopoxviruses (Grosenbach et al., 2018). However, it is unclear whether parapoxvirus infections can be effectively inhibited by tecovirimat. Thus, more efforts are needed to strengthen the development of effective drugs and vaccines.

Genistein, a natural isoflavone primarily found in soybeans and soybean-enriched products, has been shown to inhibit the replication of multiple viruses, including African swine fever virus (ASFV), Rotavirus, and Herpes simplex virus 1 (HSV-1). For example, genistein may interact with four residues of the ATP-binding site of ASFV-topoisomerase II enzyme (Asn-144, Val-146, Gly-147, and Leu-148) (Arabyan et al., 2018). The mechanism of action underlying genistein anti-rotavirus properties has been demonstrated to be involved in the upregulation of aquaporin 4 (AQP4) gene transcription via the cAMP/PKA/CREB signaling pathway (Huang et al., 2015). In addition, the anti-HSV effect of genistein was investigated, which was found to be able to inhibit HSV replication by reducing HSV-1 protein expression and HSV-2 cell-to-cell spread (Argenta et al., 2015). In the present study, we found that genistein could reduce the total yield of infectious ORFV in host cells, inhibit viral DNA replication, and viral protein synthesis. Further transcriptional analysis supported the conclusion that genistein showed efficiency in the transcription stage of ORFV life cycle. Although the transcriptional analysis of ORFV early genes (ORFV042/ORFV070), middle genes (ORFV069/ORFV072), and late genes (ORFV011/ORFV059) showed significant reduction in ORFV-infected cells treated with genistein, the result of transcriptional analysis did not completely reflect the expression levels of early, intermediate, and late proteins due to alternative splicing and post-translational modification. Whether genistein affects the viral protein expression across all stages is yet unclear, which will be a potential future direction.

Poxviruses are the cytoplasmic large DNA viruses that exclusively replicate in the cytoplasm of infected cells. The multisubunit DNA-dependent RNA polymerase (vRNAP) from poxvirus has been demonstrated to be the principal enzyme of transcription for viral gene expression in the host cytoplasm, which consists of eight subunits (e.g., Rpo147, Rpo132, Rpo35, Rpo30, Rpo22, Rpo19, Rpo18, and Rpo7) (Grimm et al., 2019, 2021). Recent studies indicated that vRNAP has emerged as a promising drug target for developing effective antiviral drugs against DNA viruses (Abduljalil and Elfiky, 2022). For example, two natural compounds (Comp289 (CNP0158693) NA and Comp441 (CNP0267280) Salpichrolide J) have been recently reported to be the drug target against the DNA-dependent RNA polymerase of human Mpox virus *in silico* (Abduljalil et al., 2023). Using molecular modeling approach, betulinic acid (BA), a pentacyclic triterpene, has also been found to be a potential inhibitor against DNA-dependent RNA polymerase (Dutt et al., 2023). It is worth noting that all the three compounds showed strong interaction profiling with the pocket residues of DdRp RPO147 throughout the simulation. A novel finding of our present study is that genistein could interact with the RPO30 subunit of ORFV DNA-dependent RNA polymerase, which is encoded by viral ORF021 gene. As an important subunit of vRNAP, RPO30 subunit has sequence similarity to mammalian protein SI1 (TFIIS), an extrinsic transcription factor required for nascent RNA cleavage by RNA polymerase II (Pol II) (Hagler and Shuman, 1993), which comprised an N-terminal domain I (NTD), a central domain, a C-terminal domain (CTD), and a unique C-terminal tail. Furthermore, RPO30 subunit was demonstrated to be firmly attached to the “funnel” domain of viral RNA polymerase RPO147 (residues 23–130), which was similar to the binding sites of TFIIS and cellular Pol II (Fish and Kane, 2002; Kettenberger et al., 2003, 2004; Mirzakhanyan and Gershon, 2017). Even though RPO30 is a homolog of TFIIS, there may be the functional and structural distinctions between viral RPO30 and TFIIS. Consequently, whether genistein interacted with similar host protein TFIIS, such as TFIIS, still needed to be validated. In our present study, using molecular modeling predictions, we found that genistein could bind to the short single-turn helical segment of the RPO30 subunit. Furthermore, the residues, Leu107 and Arg75, were confirmed to be the key sites for the binding of the RPO30 subunit of vRNAP to genistein, which might disturb the zinc ribbon domain of vRNAP or hinder the assembly of vRNAP. Although our current research demonstrated that genistein could exert the antiviral effect through the mechanism of binding of ORFV RPO30 subunit of vRNAP, the possibility that the antiviral effect of genistein was a consequence of inhibiting cellular proteins, which still could not excluded. However, it is unclear if other viral proteins or intracellular factors are involved in genistein’s antiviral effect, which will be a potential area for future research.

In conclusion, this study demonstrated that genistein could inhibit ORFV transcription by targeting the residues Leu107 and Arg75 of vRNAP RPO30 subunit, which indicated that genistein could be used as a promising therapeutic agent for ORFV infection. Additionally, the complexes vRNAP was also identified as an antiviral drug target candidate, which is expected to prevent poxvirus infection in the future. However, further investigation is required to fully understand the detailed mechanism by which genistein prevents poxvirus infection.

## Data availability statement

The original contributions presented in the study are included in the article/supplementary material, further inquiries can be directed to the corresponding authors.

## Author contributions

PL: Data curation, Writing – original draft. ZF: Software, Writing – original draft. JG: Software, Writing – original draft. LL: Software, Writing – original draft. MX: Writing – original draft. XL: Software, Writing – original draft. ZhL: Writing – original draft. YL: Software, Writing – review & editing. ZiL: Software, Writing – review & editing. HL: Software, Writing – review & editing. DS: Writing – original draft. WH: Writing – review & editing. FG: Conceptualization, Writing – review & editing. DW: Writing – review & editing. KZ: Conceptualization, Data curation, Funding acquisition, Writing – review & editing.
